# Supplementary material for: Distinct Antibody Responses to Endemic Coronaviruses Pre- and Post-SARS-CoV-2 Infection in Kenyan Infants and Mothers
Source: Viruses. 2022 Jul 12;14(7):1517. doi: 10.3390/v14071517 (PMC9323260; doi:10.3390/v14071517)
Supplement: Supplementary file 1 [file viruses-14-01517-s001.zip › viruses-1778460-supplementary.pdf]

Genus	Subgenus	Virus	% spike sequence identity with SARS-CoV-2
Betacoronavirus	*Sarbecovirus*	SARS-CoV-2	--
		SARS-CoV-1	76 [ref 17]
	Embecovirus	HCoV-OC43	30 [ref 17]
		HCoV-HKU1	29 [ref 17]
Alphacoronavirus	*Duvinacovirus*	HCoV-229E	31 [NCBI BLAST]
	Setracovirus	HCoV-NL63	31 [NCBI BLAST]

Table S1. *Virus classification and spike protein sequence identity with respect to SARS-CoV-2 spike.*

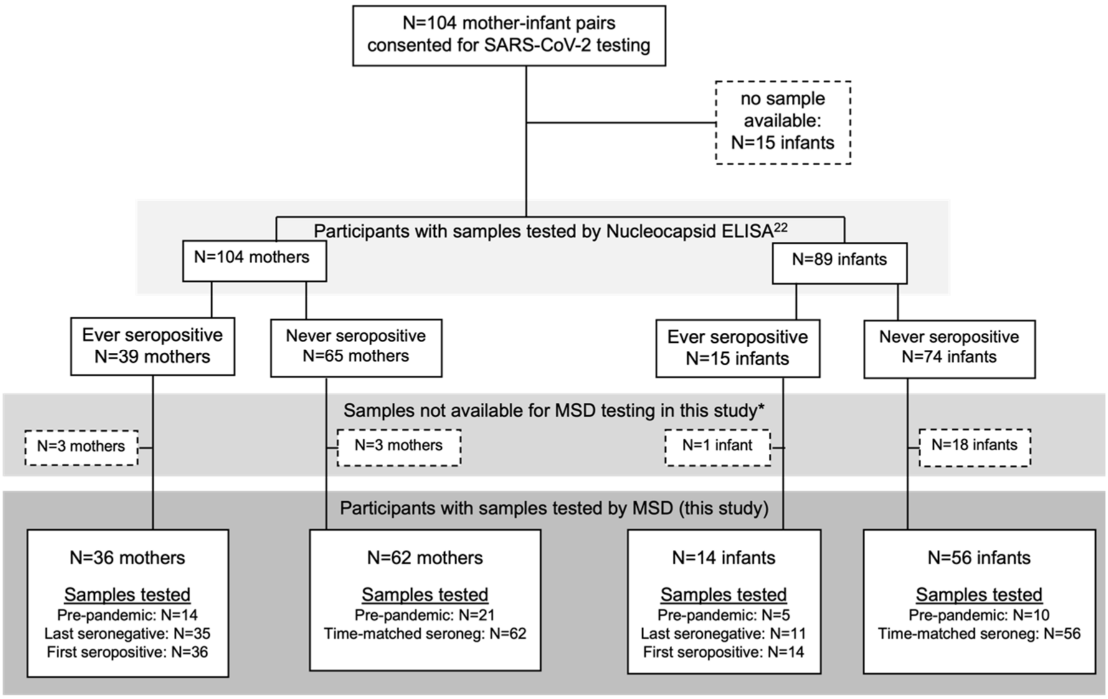

Figure S1. *Participant and sample grouping flow chart.* Consenting women and their infants were originally tested for SARS-CoV-2 by Nucleocapsid ELISA [22] and were further grouped for this sub-study as indicated. *Never seropositive participants required a "Time-matched seronegative" sample in the time window of the last negative sample from the Seroconverters (December 2019-April 2020). The collecting clinic had to pause collection during this time window due to COVID-19 restrictions and therefore infants born in early 2020 often lack a sample in this time window [22].

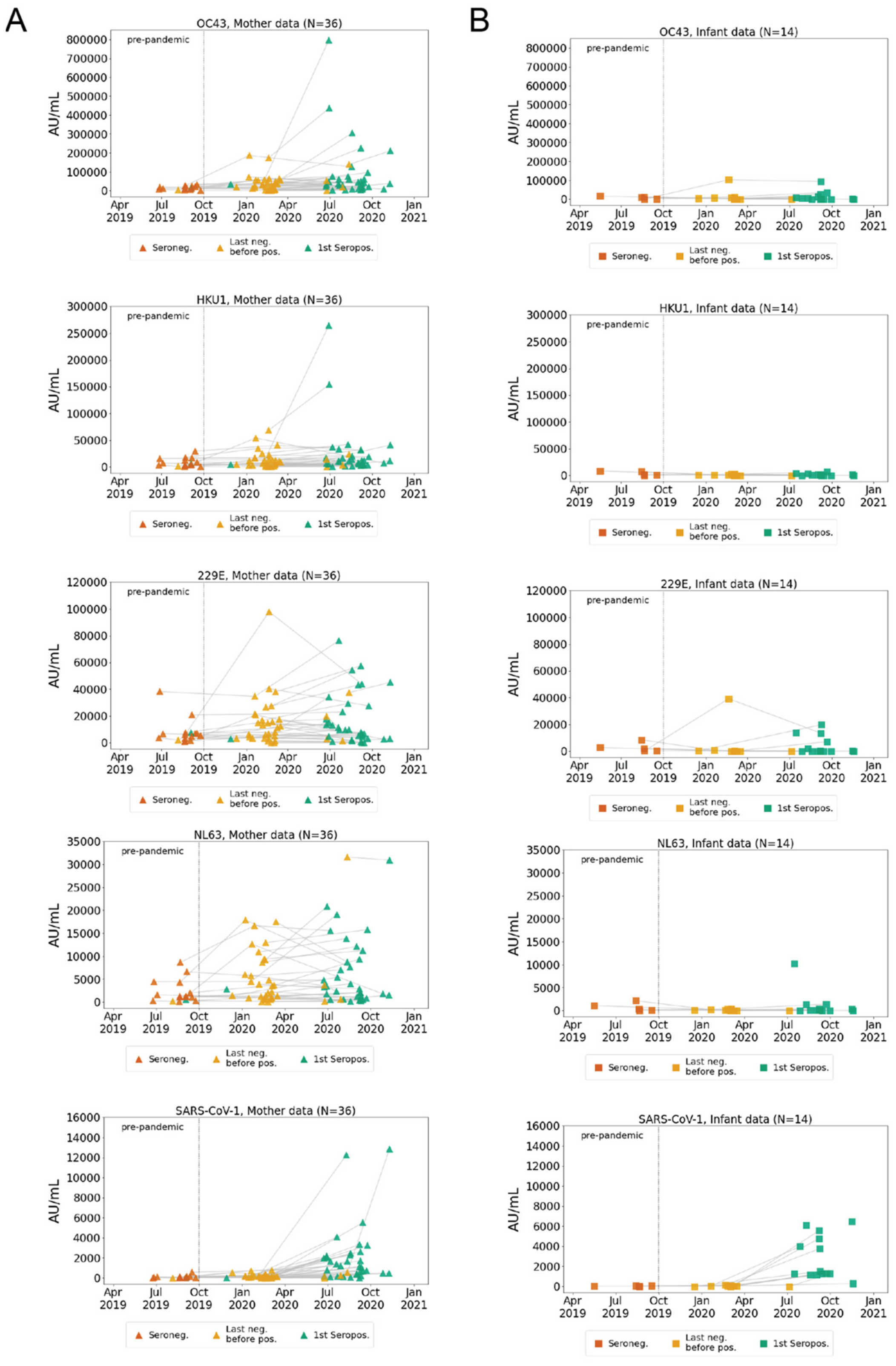

Figure S2. *Longitudinal antibody binding responses to eHCoVs and SARS-CoV-1 in individuals that eventually seroconverted to SARS-CoV-2.* IgG titers (AU/mL) over time for indicated HCoVs; left panels, eventually SARS-CoV-2 seroconverting mothers (N = 36); right panels, eventually SARS-CoV-2 seroconverting infants (N = 14).

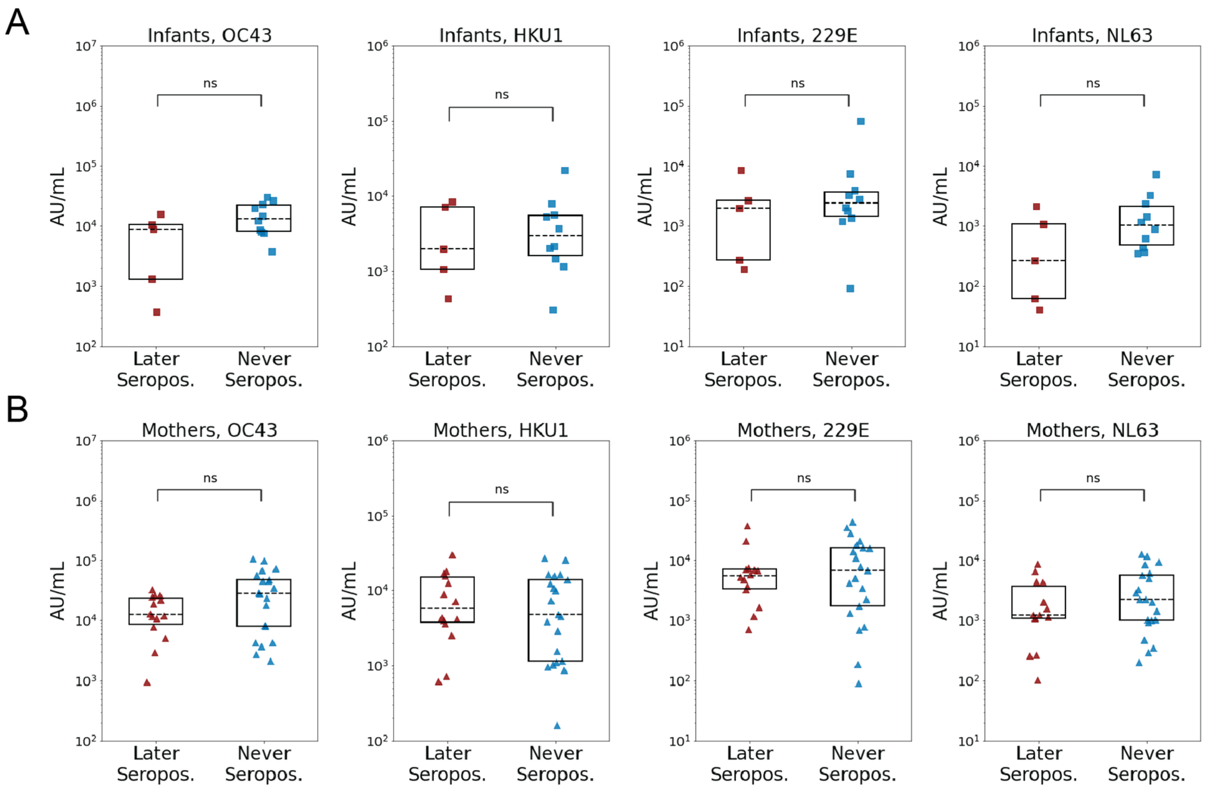

Figure S3. *Relationship between pre-pandemic eHCoV antibody titer and SARS-CoV-2 serostatus in infants and mothers.* (A) eHCoV antibody titers in infants that later became seropositive (N = 5) or were never seropositive (N = 10) for SARS-CoV-2. (B) eHCoV antibody titers in mothers that later became seropositive (N = 14) or were never seropositive (N = 21) for SARS-CoV-2. P values (A and B) were calculated using Wilcoxon rank-sum test with Bonferroni correction for multiple hypothesis testing. (ns) $P > 0.05$, (*) $P \leq 0.05$, (**) $P \leq 0.01$, (***) $P \leq 0.001$, (****) $P \leq 0.0001$.
